# Incidence, risk factors and sequelae of long/post-COVID in people with diabetes compared to people without diabetes in Germany (longcovid-diab): a study protocol

**DOI:** 10.1186/s12933-026-03222-z

**Published:** 2026-05-28

**Authors:** Tatjana Kvitkina, Maria Narres, Silke Andrich, Stefan Wilm, Andrea Icks, Heiner Claessen

**Affiliations:** 1 Institute for Health Services Research and Health Economics, German Diabetes Center, Leibniz Center for Diabetes Research at Heinrich-Heine-University Düsseldorf, Düsseldorf, Germany; 2 Institute for Health Services Research and Health Economics, Centre for Health and Society, Medical Faculty and University Hospital Düsseldorf, Heinrich-Heine-University Düsseldorf, Düsseldorf, Germany; 3 German Center for Diabetes Research, Partner Düsseldorf, Munich-Neuherberg, Germany; 4 Institute of General Practice (Ifam), Centre for Health and Society, Medical Faculty and University Hospital Düsseldorf, Heinrich-Heine-University, Düsseldorf, Germany

**Keywords:** Long/Post COVID, Incidence, Risk factors, Sequelae, Diabetes, Health insurance data, Study protocol

## Abstract

**Background:**

Several studies have shown that people with diabetes are at higher risk of experiencing a severe course of COVID-19 infection. However, it remains unclear whether diabetes per se is a risk factor for the development of Long/Post-COVID. In addition, there is a lack of nationwide, population-based studies on the epidemiology of Long/Post-COVID in people with diabetes. The aim of this project is to analyze: (1) incidence and time trends of Long/Post-COVID in people with and without diabetes between 2021 and 2023 in Germany as well as potential risk factors; (2) mortality, any hospitalization and hospitalization due to acute myocardial infarction, stroke, amputation and diabetic foot syndrome (DFS) after a Long/Post-COVID diagnosis, including an analysis of potential risk factors.

**Methods:**

This study is planned as a non-interventional longitudinal observational study based on statutory health insurance (SHI) data in Germany. The data holder is the Health Data Lab (HDL: data pool of billing data for all persons with statutory health insurance). The incidence rates of Long/Post-COVID will be estimated separately in the populations with and without diabetes and compared as corresponding relative risk. Moreover, we will analyse age- and sex-standardized mortality and hospitalization rates within 12, 24, and 36 months after a Long/Post-COVID diagnosis in the years 2021 to 2024. Potential uncertainties in diagnosing Long/Post-COVID (e.g., underreporting) will be addressed in sensitivity analyses.

**Discussion:**

The expected results will have high potential for use in epidemiological research on Long/Post-COVID, including the identification of potential risk factors in people with diabetes. The findings will serve to provide optimized healthcare for Long/Post-COVID patients with diabetes.

*Trial registration* The study has been registered in the German Clinical Trials Register with identifier DRKS00036279. Registration Date 15.05.2025.

**Graphical abstract:**

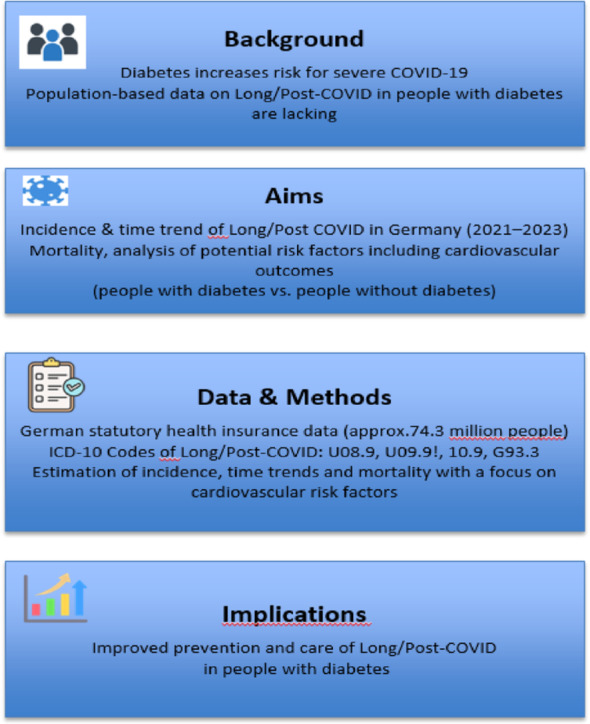

## Background

Over 39 million SARS-CoV-2-positive cases have been registered in Germany so far (RKI, August 2025), corresponding to almost 50% of the German population. It is known that some people suffer from various long-term symptoms after a COVID-19 infection [1, 2]. The symptoms can either occur in the acute phase of the disease and persist for a long time, or it is possible that new or recurring symptoms only appear over time after the infection. A wide range of heterogeneous, sometimes non-specific symptoms have been reported (respiratory, cardiovascular, neurological, and mental/psychological symptoms with varying degrees of severity). The underlying mechanisms are not yet fully understood, making the diagnosis and treatment of Long/Post-COVID difficult. In many cases, the differential diagnosis of non-specific symptoms in connection with other diseases or as a result of psychosocial stress is very complicated [2]. The definition of “Long-COVID” refers to longer-term health impairment following SARS-CoV-2 infection that persist beyond the acute phase of the disease of four weeks [3], while the “Post-COVID condition” or “Post-COVID syndrome” (WHO case definition [1]) is diagnosed when symptoms persist for at least 12 weeks or longer after the acute infection or recur after this period and cannot be explained by other factors. Given the ongoing discussion regarding the most appropriate terminology, the term “Long/Post-COVID” will be used hereafter.The diversity of symptomatic manifestations of Long/Post-COVID disease makes it difficult to define precise clinical cases and thus also to estimate their frequency [4]. The frequency of occurrence of Long/Post-COVID varies considerably depending on the population at risk, the diagnostic instruments used, the SARS-CoV-2 variant, and existing COVID-19 immunization [3]. According to a systematic review, the prevalence of Long/Post-COVID ranged from 0 to 93% with a mean of 42.1% (95% confidence interval (CI), 6.8%-7.9%) and varied depending on the definition of Long/Post-COVID, observation period after the first COVID-19 infection, and study population [5]. The incidence of Long/Post-COVID-like symptoms between 2020 and 2024 was estimated at approximately 1.4% in children and 5–6% in adults [6]. Previous studies in Germany have also shown large differences with prevalences ranging between 6.5% to 73% [7–10]. This wide range primarily reflects methodological heterogeneity.

### Comorbidities / risk factors

Several studies described the association between certain comorbidities and the development of Long/Post-COVID (e.g. systematic review by Tsampasian et al. 2023 [11]):

Conditions associated with an increased risk of Long/Post-COVID include:

- Coronary heart disease (CHD): A meta-analysis of five studies with 201,906 patients reported a significantly increased risk of Long/Post-COVID among patients with pre-existing CHD (OR 1.28; 95% CI 1.19–1.38).
- Chronic obstructive pulmonary disease (COPD): A meta-analysis of 10 studies including a total of 257,340 patients found that COPD was a risk factor associated with the development of Long/Post-COVID (OR 1.38; 95% CI 1.08–1.78).
- Bronchial asthma: A meta-analysis of 13 studies involving 639,397 patients showed that patients with asthma had a significantly higher risk of developing Long/Post-COVID (OR 1.24; 95% CI 1.15–1.35).
- Anxiety and/or depression: A pooled analysis of four studies including a total of 634,734 patients found that the risk of Long/Post-COVID was significantly increased in patients suffering from anxiety or depression (OR 1.19; 95% CI 1.02–1.40).

Other diseases (e.g. chronic kidney disease, malignancies, and autoimmune disorders) encompass broad and clinically heterogeneous conditions that are difficult to capture with sufficient specificity using ICD codes in routine data.

### Role of diabetes

Diabetes mellitus is a widespread chronic disease with a global prevalence of 11.1% among adults aged 20 to 79 [12]. Diabetes mellitus is regarded as a risk factor for severe COVID-19, as indicated by increased rates of hospitalization, intensive care admission, and mortality [13]. However, it is not yet clear whether having diabetes per se is also a risk factor for developing Long/Post-COVID. A systematic review on this topic found that 44% of the studies identified diabetes as a risk factor for Long/Post-COVID, although the strength of this association varied considerably between studies [14]. This was partly due to the quality or design of the studies, e.g., a different definition of Long/Post-COVID, study population and study size, and the length of the observation period. In addition, no studies in Germany have yet compared the incidence of Long/Post-COVID in people with diabetes to people without diabetes.

People with diabetes exhibit chronic hyperglycemia, immune dysregulation, endothelial dysfunction, and a pro-inflammatory and prothrombotic state, which may exacerbate acute infection and promote persistent post-viral pathologies. In line with these mechanisms, studies suggest that diabetes mellitus may contribute to adverse long-term outcomes following SARS-CoV-2 infection including cardiovascular conditions [15, 16]. However, the association between diabetes and Long/Post-COVID, as well as its potential cardiovascular sequelae, remains incompletely understood.

Aims of this study are: 1) to estimate the incidence and time trends of Long/Post-COVID in people with diabetes compared to those without diabetes and to analyze potential risk factors for Long/Post-COVID; 2) to analyze mortality, any hospitalization, and hospitalization due to acute myocardial infarction, stroke, amputation, and DFS within 12, 24, and 36 months after Long/Post-COVID diagnosis in people with diabetes compared to those without, and to identify potential risk factors for these conditions.

## Methods/design

### Study design and data sources

This study is planned as a non-interventional longitudinal observational study based on secondary data from the SHI in Germany. The data holder is the Health Data Lab which hosts SHI data and facilitates their analysis through secure remote processing.

The SHI data contains comprehensive information on inpatient and outpatient care with exact dates, diagnoses, prescriptions, and some sociodemographic factors (age, gender, place of residence (urban/rural)). The diabetes status of insured persons is defined according to an established algorithm based on antihyperglycemic prescriptions and outpatient and inpatient diagnoses [17]. A diagnosis of Long/Post-COVID is considered as first or incident event per se (no counting of reinfections), as this diagnosis has only been established since 2021. In light of the clinically heterogeneous presentation of Long/Post-COVID and the documented supposed inconsistencies in ICD-10 coding practices across healthcare settings, we define Long/Post-COVID on the basis of the following diagnostic coding: U08.9 (Personal history of COVID-19, unspecified; this code is used if a previous confirmed COVID-19 infection affects a person's health or leads to the use of healthcare services), U09.9! (Post-COVID-19 condition, unspecified;), U10.9 (Multisystem inflammatory syndrome associated with COVID-19, unspecified), or G93.3 (Myalgische Enzephalomyelitis/Chronisches Fatigue-Syndrom (ME/CFS). The Studies at Charité – University Medicine Berlin, which represent a major reference for research on Long/Post-COVID and ME/CFS, have shown that post-COVID syndrome can persist for more than 20 months after infection and encompass the full spectrum of post-infectious ME/CFS [18, 19]. The main features are moderate to severe fatigue and exercise intolerance, which meet the Canadian consensus criteria for ME/CFS.

The inclusion of U08.9 was based on the WHO Definition [20] and on the German national S1 guideline “Long/Post-Covid” [3]. Data from published studies also confirm widespread use of U08.9 diagnosis: For instance, the study of Ollila et al., [21] showed the distribution of codes used in Post-Covid-Syndrom: U09.9! = 12.1% and U08.9 = 47.4%. The rationale for including U08.9 was a higher sensitivity in identifying patients with probable Long/Post-COVID manifestations. The ICD code G93.3 will be taken into account only for patients with a previous COVID-19 infection. For ICD codes U09.9!,U08.9 and U10.9, a single code entry is sufficient.

In addition, the analyses will be repeated for insured persons with an outpatient or inpatient COVID-19 diagnosis (ICD: U07.1) between 2020 and 2024 as a risk population (see the following section, “Sensitivity analyses”).

## Study participants

**Table:** 

*Aim 1:*	*Participants*
Estimation of incidence and time trends of Long/Post-COVID in people with diabetes compared to those without diabetes, and analysis of potential risk factors for Long/Post COVID	All SHI-insured people in Germany (2021: 73.32 million; 2023: 74.31 million) stratified by diabetes status in the years 2021 to 2024
*Aim 2:*	
Analysis of mortality, any hospitalization, and hospitalization due to acute myocardial infarction, stroke, amputation, and DFS within 12, 24, and 36 months after Long/Post-COVID diagnosis in people with diabetes compared to those without diabetes, and identification of potential risk factors for these conditions	All SHI-insured people with and without an initial diagnosis of Long/Post-COVID, stratified according to diabetes status, will be monitored for a maximum of 36 months with regard to mortality and hospitalization due to relevant morbidity The cohort of Long/Post-COVID cases in 2021 and 2022 will be observed for up to 36 months; The cohort of Long/Post-COVID cases in 2023 will be observed for up to 12 months

### Statistical analysis

*Aim 1* The incidence rate of Long/Post-COVID will be estimated separately in the population with and without diabetes and compared as relative risks. The incidence rates will be analyzed standardized by age and sex, using the entire German population as standard population. As SHI data will be used, the person time will be calculated by summing all quarters of the insurance time of insured persons under risk. The relevant insurance time ends on the earliest of the following: the date of first diagnosis of Long/Post-COVID (index date), the end of insurance time (e.g. death) or the end of the observation period (i.e. December 31, 2023).

In addition, Poisson regression models will be used to examine the time trend of the incidence rate of Long/Post-COVID. Since only 3 years will be available, we will only compare the incidence rates of 2022 and 2023 with those of the year 2021 as reference category. Moreover, the impact of potential risk factors such as age, sex, comorbidities (CHD, COPD, asthma, obesity, depression) and region (urban vs. rural, Eastern vs. Western Germany) will be analyzed with these models. We will consider each comorbidity as well as region separately in order to explore their role independently. We will estimate incidence rates stratified by diabetes and these variables. We further plan to perform regression models adjusted for each variable and diabetes as well as models stratified by diabetes. Thus, we intend to explore whether obesity is a further confounder or effect modifier in our analysis, even in the light of probable underreporting in administrative data. To take into account over-dispersion of the outcome variable, all analyses were conducted with the de-scale adjustment [22].

*Aim 2* Age- and sex-standardized mortality and hospitalization rates will be estimated within 12, 24, and 36 months after a Long/Post-COVID diagnosis between 2021 and 2024. The data base will be a population-based cohort consisting of all insured persons with an inpatient or outpatient Long/Post-COVID diagnosis. All mortality and hospitalization rates will be calculated separately for insured people with and those without diabetes and will be compared using relative risks. In order to overcome potential confounding, Cox regression models will further be performed to investigate the impact of the aforementioned potential risk factors, as well as the year of Long/Post-COVID diagnosis. As we will conduct Cox models, we will only assess the time to first hospitalization with the date of hospital admission as index date. We will also consider death as competing risk in analyses with hospitalization as outcome.

Sensitivity analyses:

- Estimation of incidence rate of Long/Post-COVID in the population with and without diabetes and the corresponding relative risks as decribed in aim 1 using only insured persons with an outpatient or inpatient COVID-19 diagnosis as population at risk
- Repetition of analyses of aim 2 using only persons with an inpatient or outpatient Long/Post-COVID diagnosis and an additional previous COVID-19 diagnosis as population at risk
- Expansion of the outcome “Long/Post-COVID” to include comparable differential diagnoses (functional and somatoform disorders) in order to minimize the risk of bias resulting from potential underreporting of Long/Post-COVID
- Comparison of people with Long/Post-COVID and those with a differential diagnosis, stratified according to diabetes status
- Repetition of main analyses of aim 1 and aim 2 with the modified outcome.

## Discussion

Our project will contribute to gaining new insights into open questions regarding the association between diabetes and Long/Post-COVID. The results of the study will provide reliable epidemiological data on the incidence, relative risk, and time trends for Long/Post-COVID in people with diabetes which might influence health research and health policy decisions. Results will be reported in accordance with the STROSA guidelines [23].

Several expected limitations should be considered. Misclassification cannot be excluded, as the validity of coding for Long/Post-COVID conditions may vary due to differences in awareness and documentation practices across healthcare settings. Furthermore, the lack of information about the severity of symptoms could influence the risk assessment. Residual confounding cannot be excluded, as factors such as vaccination status could not be taken into account. Moreover, our analyses will not be further stratified by diabetes type since this would cause statistical power problems due to the expected small sample size.

## Data Availability

No datasets were generated or analysed during the current study.

## Abbreviations

SHI: Statutory health insurance
HDL: The health data lab
DFS: Diabetic foot syndrome
WHO: World health organization
ICD: International classification of diseases
